# On Considering Unoccupied Sites in Ecological Models

**DOI:** 10.3390/e27080798

**Published:** 2025-07-27

**Authors:** Ricardo Concilio, Luiz H. A. Monteiro

**Affiliations:** 1 Escola de Engenharia, Universidade Presbiteriana Mackenzie, São Paulo 01302-907, SP, Brazil; 2 Escola Politécnica, Universidade de São Paulo, São Paulo 05508-010, SP, Brazil

**Keywords:** cellular automaton, dynamical system, Lotka–Volterra, population dynamics, stability analysis, unoccupied sites

## Abstract

In ecosystems, spatial structure plays a fundamental role in shaping the observed dynamics. In particular, the availability and distribution of unoccupied sites—potential habitats—can strongly affect species persistence. However, mathematical models of ecosystems based on ordinary differential equations (ODEs) often neglect the explicit representation of these unoccupied sites. Here, probabilistic cellular automata (PCA) are used to reproduce two basic ecological scenarios: competition between two species and a predator–prey relationship. In these PCA-based models, unoccupied sites are taken into account. Subsequently, a mean field approximation of the PCA behavior is formulated in terms of ODEs. The variables of these ODEs are the numbers of individuals of both species and the number of empty cells in the PCA lattice. Including the empty cells in the ODEs leads to a modified version of the Lotka–Volterra system. The long-term behavior of the solutions of the ODE-based models is examined analytically. In addition, numerical simulations are carried out to compare the time evolutions generated by these two modeling approaches. The impact of explicitly considering unoccupied sites is discussed from a modeling perspective.

## 1. Introduction

Probabilistic cellular automata (PCAs) have been employed to model ecological [1,2,3], epidemiological [4,5,6], and social systems [7,8,9]. In such models, the dynamical behavior is derived from state transition rules that mimic the interactions observed in real-world scenarios. These rules can be easily implemented in digital computers; hence, the time evolution of PCA models is usually obtained from computer simulations. However, making theoretical predictions (from the PCA rules) about the spatiotemporal patterns found in these simulations is challenging. Therefore, in some studies, mean field approximations, written in terms of ordinary differential equations (ODEs) or difference equations (DEs), are concomitantly proposed [10,11,12,13,14,15,16,17,18,19,20,21,22]. These equations can be analyzed by using techniques from dynamical systems theory (DST) [23,24], allowing the long-term behavior of the PCA model to be analytically deduced. If the parameter values of the PCA model and the corresponding ODE or DE system are appropriately tuned, then both approaches yield similar results [10,11,12,18,19,20,22].

Lotka–Volterra interactions, which were introduced about 100 years ago in investigations on oscillating chemical reactions [25] and fish abundance [26], have been considered in a variety of studies, including chaotic [27] and periodic dynamics [28], chemotaxis [29], community coexistence [30], food webs [31], infection spread [32], intestinal microbiota [33], river pollution [34], self-replicating molecules [35], social behavior [36], stock markets [37], synchronization [38], technological innovation [39], vehicle-propulsion technologies [40], and weather prediction [41].

In the autonomous continuous-time version of the generalized Lotka–Volterra (LV) model, the size of the *i*th population at the time *t*, denoted by $z_{i}\left(t\right)$ (with i=1,…,n), varies according to [42,43]

(1) $$\frac{dz_{i}\left(t\right)}{dt}=z_{i}\left(t\right)\left(a_{i}+\sum_{j=1}^{n}b_{ij}z_{j}\left(t\right)\right)$$

in which $a_{i}$ is a linear rate constant (the difference between the birth rate constant and the death rate constant), $b_{ij}$ expresses the nonlinear effect of the *j*th population on the *i*th population, and *n* is the number of interacting populations. Usually, $b_{ii}<0$ in order to represent an intrapopulation competition. (Thus, if isolated, the *i*th population would follow the logistic equation; that is, $dz_{i}\left(t\right)/dt=z_{i}\left(t\right)(a_{i}-\left|b_{ii}\right|z_{i}\left(t\right)$.) The signs of $b_{ij}$ (for i≠j) specify the type of interpopulation relationship (competition, mutualism, predation, etc.). For instance, the case of anti-symmetric interactions corresponds to $b_{ij}=-b_{ji}$ [44]. LV models with time delay [45], high-order terms [46], fractional derivatives [47], stochastic noise [48], and time-varying parameters [49] have also been investigated.

Here, PCA models are proposed to represent competition and predation in an ecosystem. Such LV interactions are found, for instance, in agroforestry, in which crops and trees compete for resources (nutrients, sunlight, and water); however, trees preserve the natural enemies of the pests that attack crops [50]. Thus, there is competition between crops and trees, as well as predator–prey relationships between pests and crops, and between pest predators and pests.

Unoccupied or “empty” sites are omitted in most mathematical models of ecosystems. However, incorporating unoccupied sites can yield more realistic and predictive models, as this variable can influence the reproduction and survival of the interacting species. Indeed, ecological scenarios in which the presence of unoccupied areas plays a significant role have been documented in the literature. For instance, spatially discontinuous distributions are commonly found in landscapes inhabited by animals [51]. Topographical barriers strongly influence the home range behavior of animals and the frequency with which places are visited [52,53], even in marine environments [54]. The availability of vacant sites affects the coexistence of mutualist and non-mutualist species [55]. Spatial gaps between trees shape forest dynamics [56]. The fraction of suitable but unoccupied habitat patches determines the potential for territory expansion and resilience to extinction [57]. Destroyed habitats cannot support populations; therefore, habitat degradation leads to biodiversity loss and an increased proportion of empty areas [58], including at the boundaries between adjacent ecosystems [59]. All these examples illustrate the importance of explicitly including unoccupied sites in ecological models.

The topic discussed in the preceding paragraph invites a deeper reflection on how the spatial structure is represented in various ecological modeling approaches. It is well known that the spatial dimension is explicitly incorporated in modeling frameworks such as PCA and partial differential equations. In contrast, models based on ODEs rarely account for space, as they typically describe only the concentrations of the interacting species, disregarding the presence of empty sites. The central point of this study is to show that even in simple ecological models, such as those involving competition between only two species or classical predator–prey interactions, the inclusion of available space can significantly alter the system’s dynamics and, consequently, the predictions derived from ODE models.

In this article, the empty cells in the PCA lattice are taken into account in the mean field approximation written in terms of ODEs. These empty cells represent territories intended to be colonized by the interacting species; they are not used here for individual movement. For two interacting species, the variables are $z_{1}\left(t\right)=x\left(t\right)$, $z_{2}\left(t\right)=y\left(t\right)$, and e(t), in which e(t) denotes the number of empty cells in the PCA lattice. This modification was not considered in other mean field analyses of PCA [10,12,13,14,19]. Hence, Equation (1) is rewritten here as

(2) $$\frac{dz_{i}\left(t\right)}{dt}=z_{i}\left(t\right)\left(a_{i}e{\left(t\right)}^{\mu }+\sum_{j=1}^{n}b_{ij}z_{j}\left(t\right)e{\left(t\right)}^{\nu }\right)$$

in which μ=1 if $a_{i}>0$ and μ=0 if $a_{i}<0$, while ν=1 if $b_{ij}>0$ and ν=0 if $b_{ij}<0$. The inclusion of $e{\left(t\right)}^{\mu }$ and $e{\left(t\right)}^{\nu }$ meets the populations’ need for space to grow. In addition, $e\left(t\right)+\sum_{i=1}^{n}z_{i}\left(t\right)=\mathrm{constant}$, because the PCA lattice has fixed dimensions. This idea of including e(t) in LV models has already appeared in the literature, but it was not fully developed [21].

The remainder of this article is organized as follows. In Section 2, the PCA models are introduced. In Section 3, the equivalent ODE systems are proposed and analyzed. In Section 4, numerical simulations are presented in order to compare the results obtained from both approaches. In Section 5, the consequences of considering unoccupied sites are discussed from a mathematical modeling perspective.

## 2. PCA-Based Models

Let a two-dimensional lattice of a PCA be composed of $\sqrt{N}\times \sqrt{N}=N$ cells with periodic boundary conditions. Each cell is either occupied by a single individual or empty. Thus, for two species, at each time step *t*, each cell is in one of three states: *X* (if the cell is occupied by an individual of the first population), *Y* (if the cell is occupied by an individual of the second population), or *E* (if the cell is empty). Also, each cell contacts its eight surrounding cells. This connection structure is known as a Moore neighborhood, with a radius unit [60,61]. Throughout a computer simulation, the states of the *N* cells are updated all at once at each time step. Thus, a synchronous update scheme is employed, meaning that the states of all cells are updated simultaneously at each time step, thereby avoiding bias introduced by the update order. This approach reflects a simplified yet coherent abstraction of ecological systems, in which populations typically respond to their surroundings in parallel rather than sequentially.

As shown below, ecological interactions can be translated into probabilistic state transition rules, which define how the states of the cells composing the PCA lattice change over time. These rules are assumed to represent mutually independent stochastic events.

### 2.1. Competition

Competition means that the interaction between the species negatively affects the growth of both. For instance, in forests, two plant species can compete for the same resources. In this case, assume that the state transitions are described by

(3) $$\begin{array}{ccc} E+X & \overset{P_{1}}{⟶} & X+X \end{array}$$

(4) $$\begin{array}{ccc} X+X & \overset{P_{2}}{⟶} & E+X \end{array}$$

(5) $$\begin{array}{ccc} X+Y & \overset{P_{3}}{⟶} & E+Y \end{array}$$

(6) $$\begin{array}{ccc} E+Y & \overset{P_{4}}{⟶} & Y+Y \end{array}$$

(7) $$\begin{array}{ccc} Y+Y & \overset{P_{5}}{⟶} & E+Y \end{array}$$

(8) $$\begin{array}{ccc} Y+X & \overset{P_{6}}{⟶} & E+X \end{array}$$

in which *X*, *Y*, and *E* denote an *X* individual, a *Y* individual, and an empty cell, respectively. The notation A+B→C+D is derived from chemical kinetics, in which the plus sign denotes the encounter between molecules *A* and *B*, resulting in *C* and *D*. In this study, the letters *A* and *C* denote the state of a cell before and after its update, respectively, while letters *B* and *D* indicate the state of a neighboring cell (which remains unchanged; that is, here, B=D).

In the implementation of this PCA-based model, state transition rules are applied to each cell in a predetermined sequence. For *E*-cells, the transition rule defined by Equation (3) precedes that of Equation (6); for *X* individuals, Equation (4) is applied before Equation (5); and for *Y* individuals, Equation (7) precedes Equation (8).

The constants $P_{i}$ (i=1,…,6) are the probabilities of state transitions per time step. Observe that $P_{1}$ and $P_{4}$ are related to the birth of an individual in an empty cell; $P_{2}$ and $P_{5}$ are related to death due to intraspecific competition; and $P_{3}$ and $P_{6}$ are related to death due to interspecific competition. Thus, each *E* cell has a probability $P_{1}$ (per time step) of becoming an *X* individual and a probability $P_{4}$ (per time step) of becoming a *Y* individual (therefore, $\left(1-P_{1}\right)\left(1-P_{4}\right)$ is the probability per time step of an *E* cell remaining unoccupied). Each *X* individual has a probability $P_{2}$ of dying due to intraspecific competition and a probability $P_{3}$ of dying due to interspecific competition (hence, $\left(1-P_{2}\right)\left(1-P_{3}\right)$ is the probability per time step of an *X* individual surviving). Each *Y* individual has a probability $P_{5}$ of dying due to intraspecific competition and a probability $P_{6}$ of dying due to interspecific competition.

These six probabilities depend on the neighborhood of each cell in the following manner. Here, $P_{1}=1-e^{-k_{1}v_{x}}$, in which $k_{1}$ is a positive constant and $v_{x}$ is the number of *X* individuals within the neighborhood of an *E* cell. This formula for $P_{1}$ was chosen because $P_{1}=0$ if $v_{x}=0$ (that is, the birth of an *X* individual requires at least one *X* neighbor of an *E* cell) and $P_{1}≃1$ when $k_{1}v_{x}\gg 1$ (that is, the birth probability of an *X* individual approaches one as the product $k_{1}v_{x}$ increases). Similarly, $P_{4}=1-e^{-k_{4}v_{y}}$, in which $k_{4}$ is a positive constant and $v_{y}$ is the number of *Y* individuals within the neighborhood of an *E* cell. Also, $P_{2}=1-e^{-k_{2}v_{x}}$, in which $k_{2}>0$ and $v_{x}$ is the number of *X* individuals pertaining to the neighborhood of an *X* individual. Note that an *X* individual can die due to intraspecific competition only if $v_{x}>0$. Similarly, $P_{5}=1-e^{-k_{5}v_{y}}$, in which $k_{5}>0$ and $v_{y}$ is the number of *Y* individuals pertaining to the neighborhood of a *Y* individual. In addition, $P_{3}=1-e^{-k_{3}v_{y}}$, with $k_{3}>0$ and $v_{y}$ is the number of *Y* neighbors of an *X* individual. Observe that an *X* individual can die due to interspecific competition only if $v_{y}>0$. Similarly, $P_{6}=1-e^{-k_{6}v_{x}}$, with $k_{6}>0$ and $v_{x}$ being the number of *X* neighbors of a *Y* individual. These expressions used for computing $P_{i}$ (i=1,…,6) were inspired by other studies [22,62].

### 2.2. Predation

Predation means that the interaction between the species is beneficial for one species and detrimental to the other. For instance, in forests, caterpillars feed on leaves. Assume that *X* individuals are prey and *Y* individuals are predators. In this case, the state transitions are described by

(9) $$\begin{array}{ccc} E+X & \overset{P_{1}}{⟶} & X+X \end{array}$$

(10) $$\begin{array}{ccc} X+X & \overset{P_{2}}{⟶} & E+X \end{array}$$

(11) $$\begin{array}{ccc} X+Y & \overset{P_{3}}{⟶} & E+Y \end{array}$$

(12) $$\begin{array}{ccc} Y & \overset{P_{7}}{⟶} & E \end{array}$$

(13) $$\begin{array}{ccc} E+X+Y & \overset{P_{8}}{⟶} & Y+X+Y \end{array}$$

In this PCA-based model, state transition rules are also applied to each cell in a fixed sequential order. For *E* cells, the rule specified by Equation (13) is executed prior to that of Equation (9); for *X* individuals, Equation (10) is applied before Equation (11); and for *Y* individuals, only the transition rule defined by Equation (12) is considered.

The probabilities $P_{1}$ and $P_{2}$ have the same meaning as in the previous model. The probability $P_{3}$ is associated with the death of an *X* individual due to predation by a *Y* individual; $P_{7}$ is associated with the natural death of *Y* individuals; and $P_{8}$ is associated with the birth of a *Y* individual in an empty cell if there is an *X* neighbor (predators need prey to reproduce).

Here, $P_{3}=1-e^{-k_{3}v_{y}}$, in which $k_{3}>0$ and $v_{y}$ is the number of *Y* neighbors of an *X*-cell; $P_{7}=k_{7}$, with $0\leq k_{7}\leq 1$; and $P_{8}=1-e^{-k_{8}v_{x}v_{y}}$, in which $k_{8}>0$ and $v_{x}$ and $v_{y}$ are the numbers of *X* neighbors and *Y* neighbors for an *E* cell, respectively.

In the next section, models based on Equation (2) are proposed to represent the state transitions occurring in the PCA. In these models, geographical heterogeneities and spatial correlations related to the distribution of the species over the lattice are assumed to be negligible.

## 3. ODE-Based Models

In the ODE-based models proposed here, x(t), y(t), and e(t) represent the percentages of *X* individuals, *Y* individuals, and *E* cells at the instant *t*, respectively. The analysis of the long-term behavior of these models employs concepts taken from DST [23,24]. For instance, a steady-state solution $x\left(t\right)=x^{*}$, $y\left(t\right)=y^{*}$, and $e\left(t\right)=e^{*}$ (in which $x^{*}$, $y^{*}$, and $e^{*}$ are constants) is determined by imposing that dx(t)/dt=0, dy(t)/dt=0, and de(t)/dt=0. The stability of this solution can be inferred from the eigenvalues λ of the Jacobian matrix, obtained by linearizing the system of ODEs around it. Local asymptotical stability (that is, local convergence to this solution) requires that all eigenvalues have a negative real part. Also, the Bendixson–Dulac theorem can be applied to exclude the existence of periodic solutions in a simply connected region of the plane [23,24].

### 3.1. Competition

The mean field approximation of the PCA model presented in Section 2.1 can be written in terms of a system of ODEs as follows

(14) $$\begin{array}{ccc} \frac{dx(t)}{dt} & = & akx\left(t\right)e\left(t\right)-ax{\left(t\right)}^{2}-\delta x\left(t\right)y\left(t\right) \end{array}$$

(15) $$\begin{array}{ccc} \frac{dy(t)}{dt} & = & hqy\left(t\right)e\left(t\right)-hy{\left(t\right)}^{2}-\epsilon x\left(t\right)y\left(t\right) \end{array}$$

(16) $$\begin{array}{ccc} \frac{de(t)}{dt} & = & -akx\left(t\right)e\left(t\right)+ax{\left(t\right)}^{2}+\delta x\left(t\right)y\left(t\right)\\ & & -hqy\left(t\right)e\left(t\right)+hy{\left(t\right)}^{2}+\epsilon x\left(t\right)y\left(t\right) \end{array}$$

In these equations, ak and hq are the rate constants related to the state transitions in Equations (3) and (6) (birth); *a* and *h* are related to the state transitions in Equations (4) and (7) (intraspecific competition); and δ and ϵ are related to the state transitions in Equations (5) and (8) (interspecific competition). As expected, dx(t)/dt+dy(t)/dt+de(t)/dt=0, because x(t)+y(t)+e(t)=1. Since e(t)=1−x(t)−y(t), this system can be rewritten as

(17) $$\begin{array}{ccc} \frac{dx}{dt} & = & akx\left(1-x-y\right)-ax^{2}-\delta xy=F\left(x,y\right) \end{array}$$

(18) $$\begin{array}{ccc} \frac{dy}{dt} & = & hqy\left(1-x-y\right)-hy^{2}-\epsilon xy=G\left(x,y\right) \end{array}$$

Similar population dynamics models can be found in the literature [63,64,65].

In the state space x×y, the steady-state solution given by $x\left(t\right)=x_{i}^{*}$ and $y\left(t\right)=y_{i}^{*}$ corresponds to the equilibrium point $Q_{i}$ with coordinates $\left(x_{i}^{*},y_{i}^{*}\right)$ (evidently, $e_{i}^{*}=1-x_{i}^{*}-y_{i}^{*}$). This system has four equilibrium points, given by

$$\begin{array}{ccc} Q_{1} & = & \left(0,0\right)\\ Q_{2} & = & \left(0,\frac{q}{q+1}\right)\\ Q_{3} & = & \left(\frac{k}{k+1},0\right)\\ Q_{4} & = & \left(\frac{h(ak-\delta q)}{\theta },\frac{a(hq-\epsilon k)}{\theta }\right) \end{array}$$

with θ=ah(k+1)(q+1)−(ak+δ)(hq+ϵ).

The eigenvalues of $Q_{1}$ (extinction of both species) are $\lambda_{1}=ak>0$ and $\lambda_{2}=hq>0$; therefore, it is unstable.

The eigenvalues of $Q_{2}$ (only *Y* individuals survive) are $\lambda_{1}=\left(ak-\delta q\right)/\left(q+1\right)$ and $\lambda_{2}=-hq<0$; therefore, it is asymptotically stable if ak<δq and unstable if ak>δq.

The eigenvalues of $Q_{3}$ (only *X* individuals survive) are $\lambda_{1}=\left(hq-\epsilon k\right)/\left(k+1\right)$ and $\lambda_{2}=-ak<0$; therefore, it is asymptotically stable if hq<ϵk and unstable if hq>ϵk.

The eigenvalues of $Q_{4}$ (both species persist) are the roots of the polynomial $\lambda^{2}+\sigma_{1}\lambda +\sigma_{2}=0$, with $\sigma_{1}=ax_{4}^{*}\left(k+1\right)+hy_{4}^{*}\left(q+1\right)>0$ and $\sigma_{2}=\theta x_{4}^{*}y_{4}^{*}$. These roots have a negative real part if $\sigma_{1}>0$ and $\sigma_{2}>0$. Note that the biological conditions $x_{4}^{*}>0$ and $y_{4}^{*}>0$ imply that either θ>0 and $Q_{2}$ and $Q_{3}$ are unstable or θ<0 and $Q_{2}$ and $Q_{3}$ are asymptotically stable. However, if θ>0, then $Q_{4}$ is asymptotically stable, and if θ<0, then $Q_{4}$ is unstable (because $\sigma_{2}<0$). Therefore, when $Q_{2}$ and $Q_{3}$ are asymptotically stable, $Q_{4}$ is unstable, and when $Q_{2}$ and $Q_{3}$ are unstable, $Q_{4}$ is asymptotically stable. This result aligns with the competitive exclusion principle [66] (see also [23,24,43]).

The presence of closed orbits can be ruled out in the first quadrant of the state space x×y by employing the Bendixson–Dulac theorem with B(x,y)=1/(xy) because

(19) $$\frac{\partial (B(x,y)F(x,y))}{\partial x}+\frac{\partial (B(x,y)G(x,y))}{\partial y}=-\frac{a(k+1)}{y}-\frac{h(q+1)}{x}<0$$

In fact, the sign of ∂(BF)/∂x+∂(BG)/∂y does not change for x>0 and y>0; therefore, closed orbits (which correspond to time-periodic solutions) cannot exist in such a domain [23,24].

As an observation, if there is only a single species (for instance, if y=0), then the equilibrium points are $x^{*}=0$ (with $e^{*}=1$) and $x^{*}=k/\left(1+k\right)$ (with $e^{*}=1/\left(1+k\right)$). In this case, since ak>0, $x^{*}=k/\left(1+k\right)$ is asymptotically stable, whereas $x^{*}=0$ is unstable. Therefore, this single species would persist and occupy a fraction k/(1+k) of the territory.

### 3.2. Predation

The mean field approximation of the PCA model introduced in Section 2.2 can be written in terms of a system of ODEs as

(20) $$\begin{array}{ccc} \frac{dx(t)}{dt} & = & akx\left(t\right)e\left(t\right)-ax{\left(t\right)}^{2}-\alpha x\left(t\right)y\left(t\right) \end{array}$$

(21) $$\begin{array}{ccc} \frac{dy(t)}{dt} & = & \beta x(t)y(t)e(t)-cy(t) \end{array}$$

(22) $$\begin{array}{ccc} \frac{de(t)}{dt} & = & -akx\left(t\right)e\left(t\right)+ax{\left(t\right)}^{2}+\alpha x\left(t\right)y\left(t\right)\\ & & -\beta x(t)y(t)e(t)+cy(t) \end{array}$$

In these equations, ak and β are associated with the state transitions in Equations (9) and (13) (birth), *a* is associated with the state transition in Equation (10) (intraspecific competition), α is associated with the state transitions in Equation (11) (predation of prey), and *c* is associated with the state transition in Equation (12) (death of predators). As in the previous model, e(t)=1−x(t)−y(t). Hence, the model above can be rewritten as

(23) $$\begin{array}{ccc} \frac{dx}{dt} & = & akx\left(1-x-y\right)-ax^{2}-\alpha xy=H\left(x,y\right) \end{array}$$

(24) $$\begin{array}{ccc} \frac{dy}{dt} & = & \beta xy(1-x-y)-cy=I(x,y) \end{array}$$

Similar models appear in the literature [21,67,68].

The equilibrium points of this system are

$$\begin{array}{ccc} Q_{5} & = & \left(0,0\right)\\ Q_{6} & = & \left(\frac{k}{k+1},0\right)\\ Q_{7} & = & \left(x_{7}^{*},\frac{\beta x_{7}^{*}\left(1-x_{7}^{*}\right)-c}{\beta x_{7}^{*}}\right) \end{array}$$

in which $x_{7}^{*}$ is a positive root of

(25) $$\left(a-\alpha \right)\beta {\left(x_{7}^{*}\right)}^{2}+\alpha \beta x_{7}^{*}-c\left(ak+\alpha \right)=0$$

This equation can be written as $A{\left(x_{7}^{*}\right)}^{2}+Bx_{7}^{*}+C=0$, with A=(a−α)β, B=αβ>0, and C=−c(ak+α)<0. Its roots are $x_{7,1}^{*}=\left(-B+\sqrt{B^{2}+4A\left|C\right|}\right)/\left(2A\right)$ and $x_{7,2}^{*}=\left(-B-\sqrt{B^{2}+4A\left|C\right|}\right)/\left(2A\right)$. For A>0, only $x_{7,1}^{*}$ is a positive real root. For A<0, $x_{7,1}^{*}$ and $x_{7,2}^{*}$ are positive real roots if $B^{2}\geq 4AC$.

The eigenvalues of $Q_{5}$ (extinction of both species) are $\lambda_{1}=ak>0$ and $\lambda_{2}=-c<0$; therefore, it is unstable.

The eigenvalues of $Q_{6}$ (only the prey survives) are $\lambda_{1}=-ak<0$ and $\lambda_{2}=\left(\beta k-c{\left(k+1\right)}^{2}\right)/{\left(k+1\right)}^{2}$; therefore, it is asymptotically stable if $\beta k<c{\left(k+1\right)}^{2}$ and unstable if $\beta k>c{\left(k+1\right)}^{2}$.

The eigenvalues of $Q_{7}$ (prey and predators coexist) are the roots of the polynomial $\lambda^{2}+\rho_{1}\lambda +\rho_{2}=0$, with $\rho_{1}=a\left(k+1\right)x_{7}^{*}+\beta x_{7}^{*}y_{7}^{*}>0$ and $\rho_{2}=y_{7}^{*}\left[\left(a-\alpha \right)\beta {\left(x_{7}^{*}\right)}^{2}+c\left(ak+\alpha \right)\right]=x_{7}^{*}y_{7}^{*}\left(2Ax_{7}^{*}+B\right)$. For A>0, $\rho_{2}=x_{7,1}^{*}y_{7,1}^{*}\sqrt{B^{2}+4A\left|C\right|}>0$; therefore, $Q_{7,1}=\left(x_{7,1}^{*},y_{7,1}^{*}\right)$ is asymptotically stable. For A<0, $\rho_{2}=x_{7,1}^{*}y_{7,1}^{*}\sqrt{B^{2}-4AC}>0$ for $x_{7,1}^{*}$ and $\rho_{2}=-x_{7,2}^{*}y_{7,2}^{*}\sqrt{B^{2}-4AC}<0$ for $x_{7,2}^{*}$. Therefore, for A<0, a pair of equilibrium points ($Q_{7,1}=\left(x_{7,1}^{*},y_{7,1}^{*}\right)$ and $Q_{7,2}=\left(x_{7,2}^{*},y_{7,2}^{*}\right)$) with opposite stabilities can be created or destroyed by varying the parameter values around $B^{2}=4AC$.

In the first quadrant of the state space of this system, the Bendixson–Dulac theorem with B(x,y)=1/(xy) also excludes the existence of closed orbits. Since

(26) $$\frac{\partial (B(x,y)H(x,y))}{\partial x}+\frac{\partial (B(x,y)I(x,y))}{\partial y}=-\frac{a(k+1)}{y}-\beta <0$$

then closed orbits in the state space cannot exist for x>0 and y>0.

As a remark, if e(t) is ignored in Equations (20)–(22), then the equilibrium points are (0,0) (unstable), (k,0) (asymptotically stable if βk<c), and (c/β,a(βk−c)/(αβ)) (asymptotically stable if βk>c). Clearly, the inclusion of e(t) alters the model and the corresponding dynamics.

In the next section, numerical simulations are performed to compare the results of the PCA models and ODE models.

## 4. Numerical Simulations

**Figure 1:**
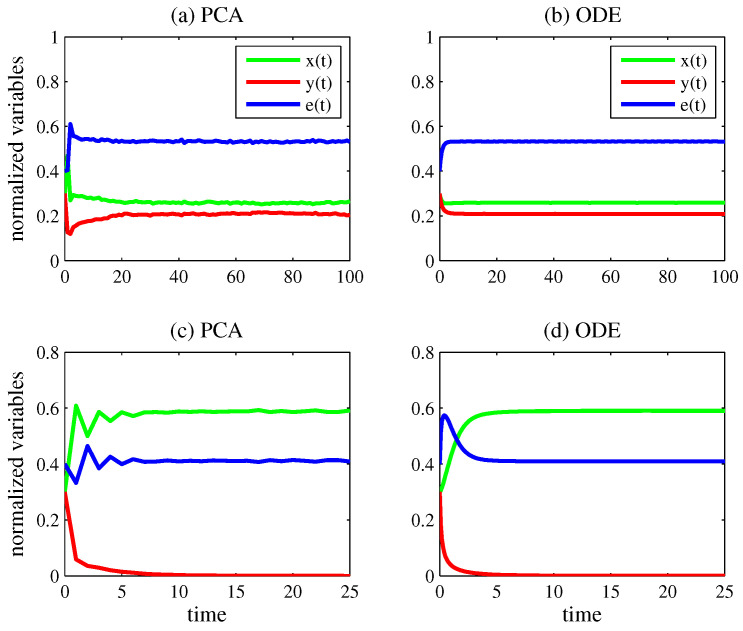
Time evolution of x(t) (green line), y(t) (red line), and e(t) (blue line). In all plots, the initial condition is (x(0),y(0),e(0))=(0.3,0.3,0.4). Case (**a**): PCA model for competition with $\sqrt{N}=200$, $k_{1}=0.2$, $k_{2}=0.1$, $k_{3}=0.05$, $k_{4}=0.3$, $k_{5}=0.1$, and $k_{6}=0.05$. By taking into account the last 20 time steps, x=0.26, y=0.21, and e=0.53 on average. Case (**b**): ODE model for competition with ak=1.22, a=2.40, δ=0.14, hq=1.18, h=2.82, and ϵ=0.15. As in the PCA simulation, x(t)→0.26, y(t)→0.21, and e(t)→0.53 for t→∞. In (**a**,**b**), the two species persist. Case (**c**): PCA model for competition with $\sqrt{N}=200$, $k_{1}=0.5$, $k_{2}=0.2$, $k_{3}=0.2$, $k_{4}=0.2$, $k_{5}=0.2$, and $k_{6}=0.1$. By taking into consideration the last 5 time steps, x=0.59, y=0, and e=0.41 on average. Case (**d**): ODE model for competition with ak=1.47, a=1.02, δ=0.84, hq=0.36, h=21.6, and ϵ=0.77. As in the PCA simulation, x(t)→0.59, y(t)→0, and e(t)→0.41 for t→∞. In (**c**,**d**), only the first species survives.

Figure 1 presents the time evolution of x(t) (green line), y(t) (red line), and e(t) (blue line) when considering the models for competition. In all plots, the initial conditions are (x(0),y(0),e(0))=(0.3,0.3,0.4) and $\sqrt{N}=200$ (thus, the lattice is composed of N= 40,000 cells).

In case (a), the PCA model was simulated with $k_{1}=0.2$, $k_{2}=0.1$, $k_{3}=0.05$, $k_{4}=0.3$, $k_{5}=0.1$, and $k_{6}=0.05$. Figure 1 also presents the numerical solution of Equations (14)–(16) using the fourth-order Runge–Kutta method [69] with an integration time step of 0.01. The parameter values of the ODE model for competition were estimated from the PCA simulation by

(27) $$\begin{array}{ccc} ak & = & \frac{\Delta_{3}x\left(t\right)}{x\left(t\right)e\left(t\right)N^{2}\Delta t} \end{array}$$

(28) $$\begin{array}{ccc} a & = & \frac{\Delta_{4}x\left(t\right)}{x{\left(t\right)}^{2}N^{2}\Delta t} \end{array}$$

(29) $$\begin{array}{ccc} \delta & = & \frac{\Delta_{5}x\left(t\right)}{x\left(t\right)y\left(t\right)N^{2}\Delta t} \end{array}$$

(30) $$\begin{array}{ccc} hq & = & \frac{\Delta_{6}y\left(t\right)}{y\left(t\right)e\left(t\right)N^{2}\Delta t} \end{array}$$

(31) $$\begin{array}{ccc} h & = & \frac{\Delta_{7}y\left(t\right)}{y{\left(t\right)}^{2}N^{2}\Delta t} \end{array}$$

(32) $$\begin{array}{ccc} \epsilon & = & \frac{\Delta_{8}y\left(t\right)}{x\left(t\right)y\left(t\right)N^{2}\Delta t} \end{array}$$

in which $\Delta_{3}x\left(t\right)$, $\Delta_{4}x\left(t\right)$, $\Delta_{5}x\left(t\right)$, $\Delta_{6}y\left(t\right)$, $\Delta_{7}y\left(t\right)$, and $\Delta_{8}y\left(t\right)$ are the numbers of state transitions in Equations (3), (4), (5), (6), (7) and (8) per time step Δt found in the PCA lattice for $T_{1}<t\leq T_{2}$, respectively. In case (b), $T_{1}=80$ and $T_{2}=100$; therefore, the average values of these parameters were computed by taking into account the last 20 time steps of the PCA simulation (when the system already reached its long-term behavior). In case (b), ak=1.22, a=2.40, δ=0.14, hq=1.18, h=2.82, and ϵ=0.15. With these parameter values, x(t)→0.26, y(t)→0.21, and e(t)→0.53 for t→∞ in the ODE model. These are the coordinates of the equilibrium point $Q_{4}$, which represents coexistence. These are also the average values of the normalized variables computed in the last 20 time steps of the PCA simulation.

In case (c), the PCA model was simulated with $k_{1}=0.5$, $k_{2}=0.2$, $k_{3}=0.2$, $k_{4}=0.2$, $k_{5}=0.2$, and $k_{6}=0.1$. By taking $T_{1}=20$ and $T_{2}=25$ (the last 5 time steps), x=0.59, y=0, and e=0.41 on average in the lattice. The parameter values of the ODE model in case (d) were ak=1.47, a=1.02, δ=0.84, hq=0.36, h=21.6, and ϵ=0.77. Consequently, the normalized variables approach the same values found in the PCA simulation. In cases (c) and (d), only the first species survived, which corresponds to convergence to the equilibrium point $Q_{3}$.

**Figure 2:**
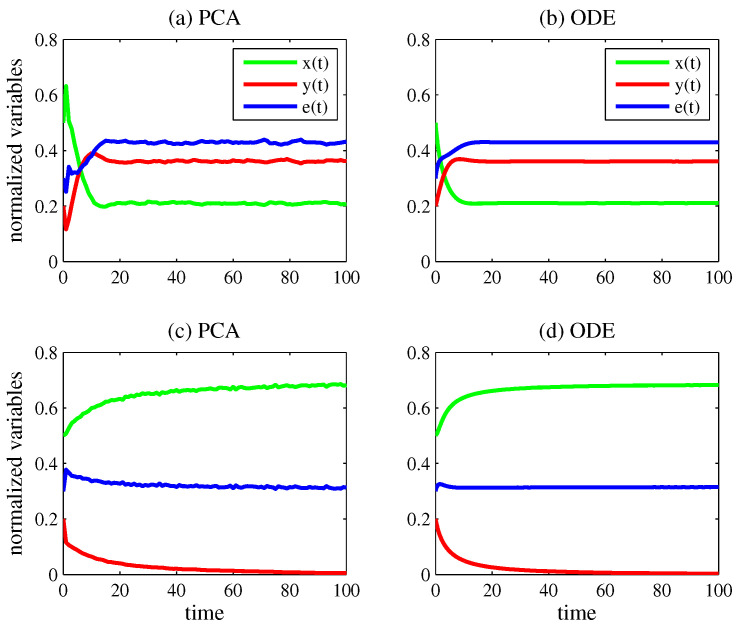
Time evolution of x(t) (green line), y(t) (red line), and e(t) (blue line). In all plots, the initial condition is (x(0),y(0),e(0))=(0.5,0.2,0.3). Case (**a**): PCA model for predation with $\sqrt{N}=200$, $k_{1}=0.3$, $k_{2}=0.09$, $k_{3}=0.09$, $k_{7}=0.3$, and $k_{8}=0.1$. By considering the last 20 time steps, x=0.21, y=0.36, and e=0.43 on average. Case (**b**): ODE model for predation with ak=0.85, a=0.86, α=0.51, c=0.30, and β=3.32. As a consequence, x(t)→0.21, y(t)→0.36, and e(t)→0.43 for t→∞. In (**a**,**b**), the two species coexist. Case (**c**): PCA model for predation with $\sqrt{N}=200$, $k_{1}=0.4$, $k_{2}=0.09$, $k_{3}=0.09$, $k_{7}=0.9$, and $k_{8}=0.1$. By taking into consideration the last 20 time steps, x=0.69, y=0, and e=0.31 on average. Case (**d**): ODE model for predation with ak=1.24, a=0.57, α=0.35, c=0.92, and β=4.19. As in the PCA simulation, x(t)→0.69, y(t)→0, and e(t)→0.31 for t→∞. In (**c**,**d**), only the prey survives.

Figure 2 exhibits the time evolution of x(t) (green line), y(t) (red line), and e(t) (blue line) using the models for predation. In all plots, the initial condition is (x(0),y(0),e(0))=(0.5,0.2,0.3), and the lattice is composed of N=40,000 cells.

In case (a), the PCA model was simulated with $k_{1}=0.3$, $k_{2}=0.09$, $k_{3}=0.09$, $k_{7}=0.3$, and $k_{8}=0.1$. Figure 2 also exhibits the time evolution of x(t), y(t), and e(t) obtained from the numerical integration of Equations (20)–(22). The parameter values of the ODE model for predation were estimated from the PCA simulation by

(33) $$\begin{array}{ccc} ak & = & \frac{\Delta_{9}x\left(t\right)}{x\left(t\right)e\left(t\right)N^{2}\Delta t} \end{array}$$

(34) $$\begin{array}{ccc} a & = & \frac{\Delta_{10}x\left(t\right)}{x{\left(t\right)}^{2}N^{2}\Delta t} \end{array}$$

(35) $$\begin{array}{ccc} \alpha & = & \frac{\Delta_{11}x\left(t\right)}{x\left(t\right)y\left(t\right)N^{2}\Delta t} \end{array}$$

(36) $$\begin{array}{ccc} c & = & \frac{\Delta_{12}y\left(t\right)}{y(t)N\Delta t} \end{array}$$

(37) $$\begin{array}{ccc} \beta & = & \frac{\Delta_{13}y\left(t\right)}{x\left(t\right)y\left(t\right)e\left(t\right)N^{3}\Delta t} \end{array}$$

in which $\Delta_{9}x\left(t\right)$, $\Delta_{10}x\left(t\right)$, $\Delta_{11}x\left(t\right)$, $\Delta_{12}y\left(t\right)$, and $\Delta_{13}y\left(t\right)$ are the numbers of state transitions in Equations (9), (10), (11), (12) and (13) per time step Δt found in the PCA lattice for $T_{1}<t\leq T_{2}$, respectively. In case (b), the parameter values of Equations (20)–(22) computed from the PCA simulation by taking $T_{1}=80$ and $T_{2}=100$ (the last 20 time steps) were ak=0.85, a=0.86, α=0.51, c=0.30, and β=3.32. In the ODE plot, (x(t),y(t),e(t))→(0.21,0.36,0.43) for t→∞, which are the coordinates of the equilibrium point $Q_{7,1}$. In the PCA plot, these are the average values of the normalized variables for 80<t≤100. Thus, in (a) and (b), both species persist.

In case (c), the PCA model was simulated with $k_{1}=0.4$, $k_{2}=0.09$, $k_{3}=0.09$, $k_{7}=0.9$, and $k_{8}=0.1$. By considering $T_{1}=80$ and $T_{2}=100$, x=0.69, y=0, and e=0.31 on average in the lattice. The parameter values of the ODE model in case (d) were ak=1.24, a=0.57, α=0.35, c=0.92, and β=4.19; hence, x(t)→0.69, y(t)→0, and e(t)→0.31 for t→∞. In (c) and (d), only the prey survives, which corresponds to convergence to the equilibrium point $Q_{6}$.

Note the good agreement of the long-term behavior in the plots obtained from both approaches. This agreement held for virtually any initial condition. Naturally, when two or more asymptotically stable equilibrium points coexist, the initial condition determines which equilibrium point is reached. For instance, in the case in which both species persist in the competition scenario, the system converges to the equilibrium $Q_{4}$, as illustrated in Figure 1a,b. This convergence occurs from any initial condition that does not coincide with the unstable equilibrium points $Q_{1}$, $Q_{2}$, or $Q_{3}$. In the case in which only one species persists, the initial condition determines whether the system converges to $Q_{2}$ or $Q_{3}$. In Figure 1c,d, the chosen initial condition leads the system to converge to $Q_{3}$.

Also note that the values of the constants $k_{i}$ (i=1,…,8) used in the PCA models affect the parameter values of the ODE systems, which can be estimated from numerical simulations (or even ecological data) by using Equations (27)–(37).

As a final remark, the low-amplitude oscillations in the asymptotic dynamics observed in Figure 2a may arise from the stochasticity of the PCA model, particularly when the eigenvalues of the steady-state solution of the corresponding ODE model have imaginary parts. This phenomenon has already been reported in the literature [70]. In fact, in Figure 2b, the eigenvalues are −0.31±0.27i (and the standard deviation of the mean asymptotic values found in Figure 2a was about 15%). In contrast, the eigenvalues of the steady-state solution in Figure 1b are real (−0.57 and −1.20), and this may explain why the oscillations in Figure 1a are smaller than those in Figure 2a (the standard deviation of the mean asymptotic values found in Figure 1a was about 7%).

## 5. Discussion and Conclusions

Spatial patch models have been developed to predict the abundance, distribution, and diversity of species in biomes [1,2,3,71,72,73]. This topic is relevant because biodiversity affects ecosystem resilience and carbon sequestration, which influence the climate change we are currently facing. Competition and predation are two key interactions that shape the dynamics in ecosystems. Here, these interactions were represented by PCA state transition rules. When the species are homogeneously distributed over the space, the variation in the size of each population can be described by an LV model written as a set of ODEs. Also, in this article, the variable e(t) was considered in the ODE-based models to take into account the empty cells in the PCA lattice. This specific modification of the LV model was used in reference [21] in Equation (2) for the term with the constant $a_{i}$ but not the term with the constant $b_{ij}$.

In the case of competition, either both species coexist or only one of the two species survives. Note that the inclusion of e(t) did not alter the type of nonlinearity of the ODE system; that is, $dx/dt=x-x^{2}-xy$ and $dy/dt=y-y^{2}-xy$ by taking into account e(t) or not (that is, either from Equation (1) or from Equation (2), assuming that all parameters values are equal to 1 and x+y+e=1).

In the case of predation, either both species coexist or only the prey population persists. In the ODE system, however, the inclusion of e(t) did alter the nonlinearity because $dx/dt=x-x^{2}-xy$ and dy/dt=−y+xy by ignoring e(t), but $dx/dt=x-x^{2}-xy$ and $dy/dt=-y+xy-x^{2}y-xy^{2}$ by considering e(t). Note that the terms $-x^{2}y-xy^{2}$ appear in dy/dt only if e(t) is taken into consideration. The presence of these terms affects, for instance, the number and location of equilibrium points, as well as their stability conditions (as shown at the end of Section 3).

Therefore, taking into consideration the variable e(t) can qualitatively modify the vector field of the corresponding ODE system, which can impact its dynamical behavior. This finding leads to the following proposition: Theoretical investigations of ecosystem dynamics should explicitly consider the variable e(t), even when the homogeneous mixing assumption holds. Including of this variable can enhance our understanding of the spatial structures of ecosystems and improve the realism of predictions.

## Data Availability

This manuscript has no associated data.
